# Monitoring Information-Seeking Patterns and Obesity Prevalence in Africa With Internet Search Data: Observational Study

**DOI:** 10.2196/24348

**Published:** 2021-04-29

**Authors:** Olubusola Oladeji, Chi Zhang, Tiam Moradi, Dharmesh Tarapore, Andrew C Stokes, Vukosi Marivate, Moinina D Sengeh, Elaine O Nsoesie

**Affiliations:** 1 Department of Global Health, School of Public Health Boston University Boston, MA United States; 2 Department of Computer Science Boston University Boston, MA United States; 3 Department of Computer Science University of Pretoria Pretoria South Africa; 4 Directorate of Science, Technology and Innovation Freetown Sierra Leone

**Keywords:** obesity, overweight, Africa, chronic diseases, hypertension, digital phenotype, infodemiology, infoveillance

## Abstract

**Background:**

The prevalence of chronic conditions such as obesity, hypertension, and diabetes is increasing in African countries. Many chronic diseases have been linked to risk factors such as poor diet and physical inactivity. Data for these behavioral risk factors are usually obtained from surveys, which can be delayed by years. Behavioral data from digital sources, including social media and search engines, could be used for timely monitoring of behavioral risk factors.

**Objective:**

The objective of our study was to propose the use of digital data from internet sources for monitoring changes in behavioral risk factors in Africa.

**Methods:**

We obtained the adjusted volume of search queries submitted to Google for 108 terms related to diet, exercise, and disease from 2010 to 2016. We also obtained the obesity and overweight prevalence for 52 African countries from the World Health Organization (WHO) for the same period. Machine learning algorithms (ie, random forest, support vector machine, Bayes generalized linear model, gradient boosting, and an ensemble of the individual methods) were used to identify search terms and patterns that correlate with changes in obesity and overweight prevalence across Africa. Out-of-sample predictions were used to assess and validate the model performance.

**Results:**

The study included 52 African countries. In 2016, the WHO reported an overweight prevalence ranging from 20.9% (95% credible interval [CI] 17.1%-25.0%) to 66.8% (95% CI 62.4%-71.0%) and an obesity prevalence ranging from 4.5% (95% CI 2.9%-6.5%) to 32.5% (95% CI 27.2%-38.1%) in Africa. The highest obesity and overweight prevalence were noted in the northern and southern regions. Google searches for diet-, exercise-, and obesity-related terms explained 97.3% (root-mean-square error [RMSE] 1.15) of the variation in obesity prevalence across all 52 countries. Similarly, the search data explained 96.6% (RMSE 2.26) of the variation in the overweight prevalence. The search terms yoga, exercise, and gym were most correlated with changes in obesity and overweight prevalence in countries with the highest prevalence.

**Conclusions:**

Information-seeking patterns for diet- and exercise-related terms could indicate changes in attitudes toward and engagement in risk factors or healthy behaviors. These trends could capture population changes in risk factor prevalence, inform digital and physical interventions, and supplement official data from surveys.

## Introduction

Globally, obesity and overweight are the fifth leading cause of death, associated with at least 2.8 million adult deaths each year [1,2]. In Africa, the burden of obesity and overweight has increased significantly over the last two decades [3-6]. Among sub-Saharan African women, the prevalence of obesity increased by 12% between 1975 and 2016, while the prevalence of overweight increased by 24% [7-9]. Among men, obesity prevalence increased by 5%, while overweight prevalence increased by 15% in the same period [7-9].

Insufficient exercise and unhealthy diets (partly due to a nutrition transition from nutrient-dense foods to energy-dense foods) coupled with tobacco use and excessive alcohol consumption (factors predominantly associated with an urban lifestyle) are to blame for the increase in noncommunicable disease burden in Africa [10,11]. Specifically, urbanization and related economic advancements including higher income, higher education, and higher socioeconomic status have been associated with higher obesity prevalence [12-16]. Aging, cultural norms (eg, in some cultures female fatness symbolizes beauty, prosperity, and fertility), and television viewing habits have also correlated with increasing obesity prevalence [16-20].

Persons who are obese or overweight are at a higher risk of developing other medical conditions including hypertension, cardiovascular disease, type 2 diabetes, and stroke [21-24]. Joubert et al [25] noted that 68% of hypertensive disease, 38% of ischemic heart disease, 78% of type 2 diabetes, and 45% of ischemic stroke among adults in South Africa were due to obesity. The burden of obesity-associated noncommunicable diseases is expected to continue to increase in sub-Saharan African countries. Data suggest that millions of people living with diabetes in sub-Saharan Africa are unaware of their status and many lack access to necessary information and medications [4,26-29]. Furthermore, obesity-related diseases have been associated with an increased risk of severe COVID-19 disease [30].

The rise in prevalence of noncommunicable diseases in Africa creates new challenges that many health care systems are not currently equipped to manage. Furthermore, the lack of high-quality data also creates a barrier in quantifying public health needs and addressing the impact of diseases [31]. This data limitation includes a substantial gap in the standard and availability of health data, especially where health information is not digitized or comprehensive [31].

Usually, data on behavioral risk factors are collected through surveys, which can be costly and capture only a single time point. In contrast, digital data from internet sources can capture timely changes in attitudes toward and engagement in risky behaviors. While computational and statistical approaches have been successfully used to process data from digital sources for monitoring infectious disease reports and chronic disease risk factors, few studies have focused on Africa [31-43]. As more people in Africa use internet platforms and mobile phones for seeking and sharing information, it is important to understand how behavioral data shared on digital platforms can be used to support and develop timely disease and risk factor surveillance platforms. Here, we assess how diet- and exercise-related searches submitted on an internet search engine can be used for monitoring information-seeking patterns and obesity prevalence in 52 African countries.

## Methods

### Data Collection

Search data were collected for 108 search terms (Multimedia Appendix 1) from Google application programming interfaces. The search terms included terms related to chronic diseases, risk factors, diet, and physical activity. To generate a comprehensive list of terms, we used the Google Trends website [44] to identify terms that had similar search trends for chronic diseases and their associated risk factors. We collected the yearly search volume for each country from 2010 to 2016 for 52 countries in English [45]. Google normalizes the search volume for each term relative to the search activity in the country and the specific time period. Two countries (South Sudan and Sudan) were excluded because obesity prevalence estimates were unavailable for these countries.

We also downloaded age-standardized obesity and overweight prevalence estimates for adults aged 18 years and older from 2010 to 2016 from the World Health Organization (WHO) website [46,47]. These estimates were obtained using data from population-based studies on cardiometabolic risk factors, multicountry and national measurement surveys, as well as the WHO STEPwise approach to surveillance (STEPS) surveys for estimating BMI [48]. Overweight was defined as a BMI >25 kg/m^2^ and obese was defined as a BMI ≥30 kg/m^2^ [49]. The reported credible intervals (CIs) for the estimates represented the 2.5th and 97.5th percentiles of the posterior distributions.

### Machine Learning Methods

We used machine learning methods to identify search patterns that were associated with changes in obesity and overweight prevalence across African countries. Specifically, we employed support vector machine (SVM), random forest (RF), gradient boosting, and Bayes generalized linear model (GLM). The machine learning methods were selected to assess a broad range of approaches from decision tree methods, kernel-based approaches, and least squares regression methods. We implemented these methods using the SuperLearner package in R [50,51], which generates estimates for each individual method and an ensemble of the methods.

RF regression is an extension of bootstrap aggregating (“bagging”). It involves the construction of de-correlated decision trees, which are averaged to reduce the variance of the prediction function. Trees are preferred candidates for bagging because they capture the complex interaction structures in the data and have relatively low bias if grown deep. Since each generated tree in bagging is identically distributed, the average of B such trees is the same as the likelihood of any one of the trees. The gradient boosting algorithm also involves the generation of ensembles of predictive trees. However, trees are built using the gradient boosting approach, which involves a sequential iterative fitting procedure to reduce bias by assigning higher weights to poorly fit samples and optimization via a loss function. An advantage of the gradient boosting algorithm is that nonlinearities and interactions do not need to be explicitly specified.

In contrast, SVM regression is similar to multiple linear regression when the relationship between X and y is linear:  y = ƒ(x)  = W · X + b. However, SVM regression involves the application of kernel functions (eg, gaussian, polynomial, radial basis, and sigmoid kernel) to model nonlinearity between X and y. The SVM regression model parameters are selected to minimize an epsilon-insensitive cost function. The model parameters were selected by applying cross-validation to the training data.

Lastly, Bayes GLMs are a class of GLMs that are a generalization of linear regression models such that the distribution of the dependent variable is of the exponential family (eg, gaussian, poisson, binomial, categorical, multinomial, or beta). In the Bayesian approach, inferences are based on the posterior distribution, prior knowledge is captured quantitatively through the prior distribution, and the data are represented through the likelihood function [52,53]. Two advantages of Bayesian models include the incorporation of domain knowledge via the prior and uncertainty quantification via the posterior distribution.

### Data Analysis

First, we estimated the Pearson correlation coefficient (*r*) between the search data and obesity and overweight prevalence across Africa from 2010 to 2016. Next, we excluded all search terms that had zero variance (ie, 20 search terms) and search terms not significantly correlated with obesity/overweight prevalence at a significance level of *P*<.05. Additionally, because there were zero reported searches for some terms in some countries, we excluded all terms with less than 50% of observations greater than zero, implying that only the most significant and comprehensive variables were used in the modeling. We then fitted separate models to estimate obesity and overweight prevalence using the search data. The coefficient of determination (*R*^2^) and root-mean-square error (RMSE) were used to assess the model fit. The out-of-sample estimation involved splitting the data into 2 sets: data from 2010 to 2014 were used to train the model, while data from 2015 to 2016 were used to evaluate the model. In machine learning, the data used to train the model are usually different from the data used to validate it. The training data are used to fit the model (ie, train the algorithm to identify patterns) and the evaluation data are used to assess the predictive performance of the fitted model by comparing the model estimates to true values. The aim is to allow the model to be generalizable to future sets of data. However, in the absence of future data, the evaluation data are used. We also report the correlation between the out-of-sample predictions and WHO-estimated obesity and overweight prevalence. The following R packages were used: SuperLearner, randomForest, kernlab, and arm [51,54].

## Results

### Information-Seeking Patterns

Some countries had sparse or no data for some of the search terms. Search patterns were similar for several of the terms: lose weight and weight (*r*=0.93, 95% CI 0.91-0.94), diet and weight (*r*=0.92, 95% CI 0.90-0.93), diet and weight loss (*r*=0.89, 95% CI 0.87-0.91), food and weight (*r*=0.88, 95% CI 0.85-0.90), food and weight loss (*r*=0.86, 95% CI 0.83-0.88), breakfast and diet (*r*=0.85, 95% CI 0.82-0.87), weight and ginger (*r*=0.84, 95% CI 0.81-0.87), weight and breakfast (*r*=0.83, 95% CI 0.80-0.86), weight loss and weight gain (*r*=0.83, 95% CI 0.79-0.86), exercise and food (*r*=0.81, 95% CI 0.77-0.84), ginger and weight loss (*r*=0.81, 95% CI 0.77-0.84), weight loss and fasting (*r*=0.81, 95% CI 0.77-0.84), gym and diet (*r*=0.81, 95% CI 0.77-0.84), lose weight and food (*r*=0.81, 95% CI 0.77-0.84), lose weight and gym (*r*=0.81, 95% CI 0.77-0.84), and food and ginger (*r*=0.80, 95% CI 0.75-0.83). Most of these associations were between terms that capture the same underlying intention. For instance, someone searching for information on how to lose weight might also search for gym, diet, or weight loss plans.

**Figure 1:**
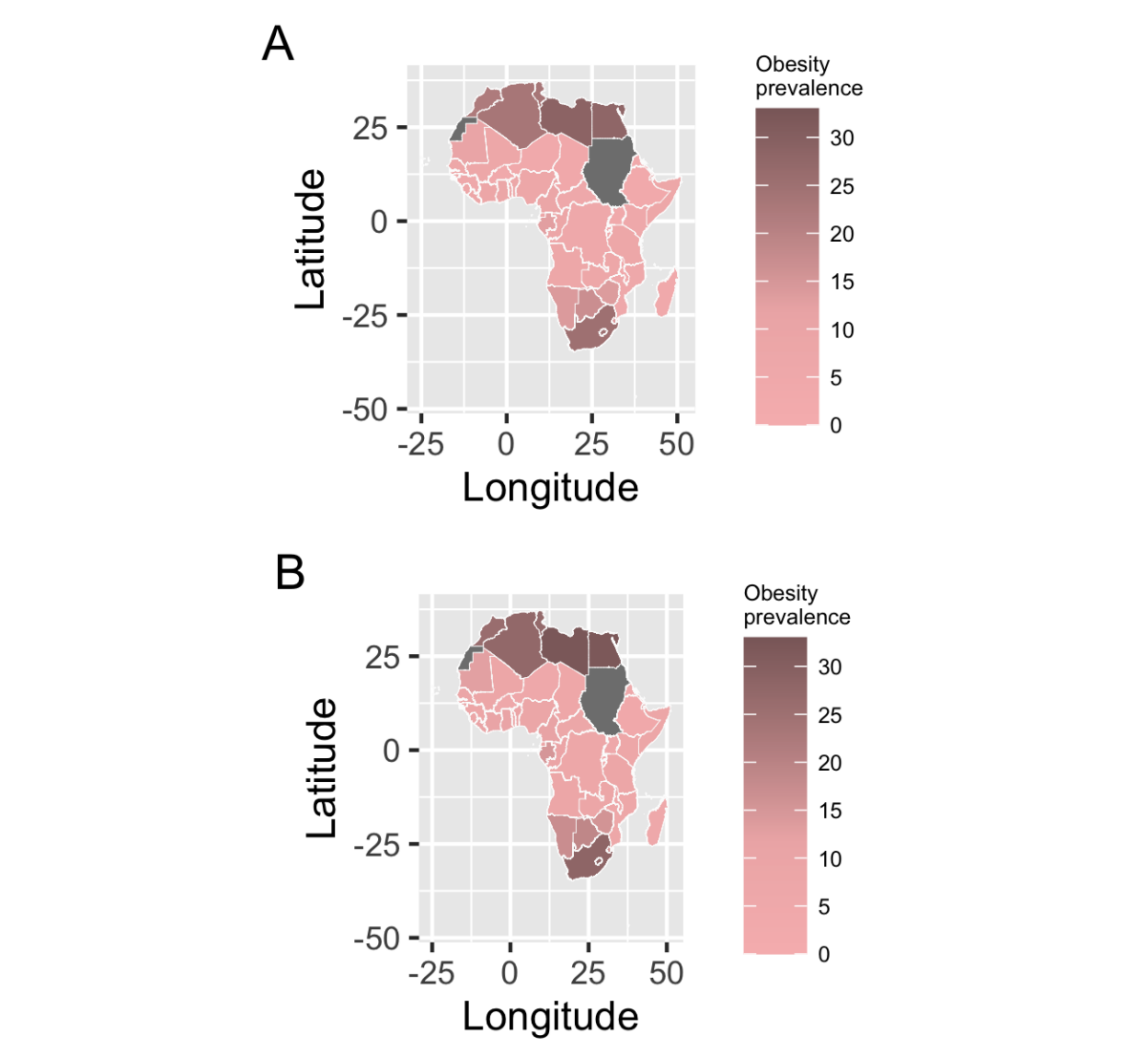
Estimated adult obesity prevalence in Africa from the World Health Organization in (A) 2010 and (B) 2016.

**Figure 2:**
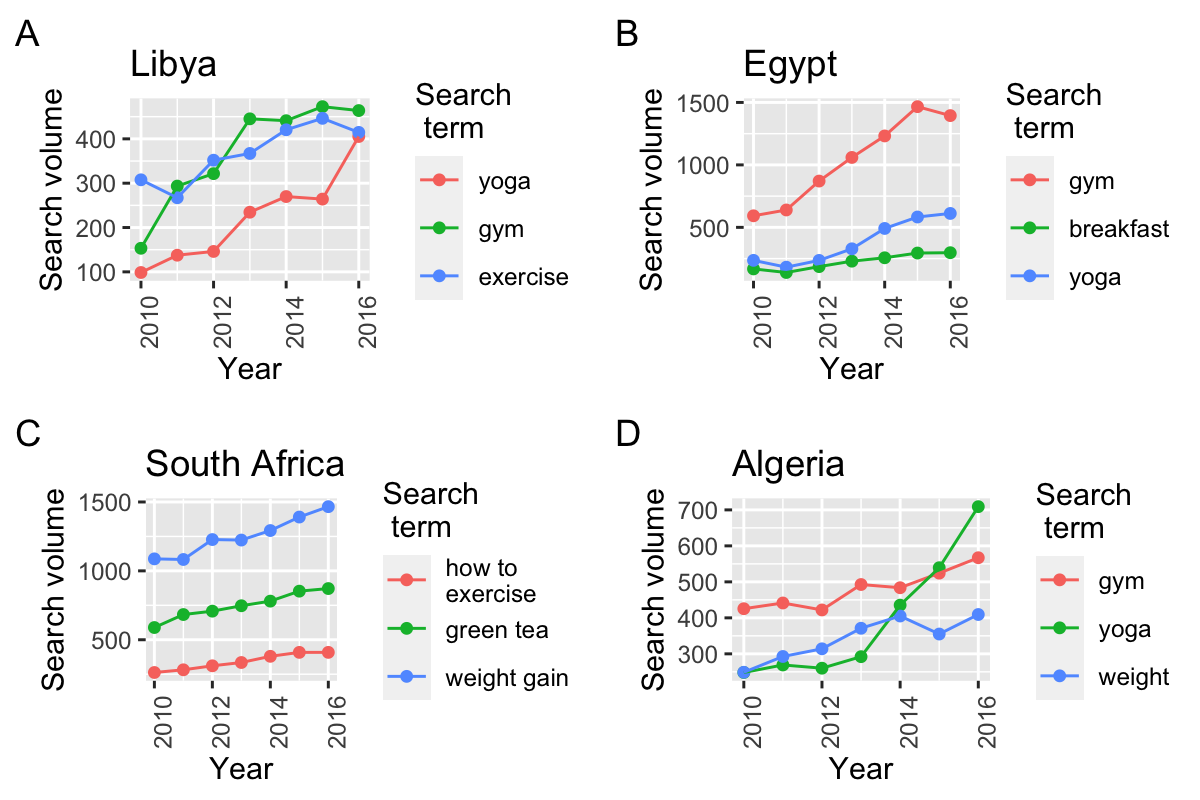
Search trends for the terms most correlated with obesity and overweight prevalence estimates from the World Health Organization for countries with the highest obesity and overweight prevalence in Africa: (A) Libya, (B) Egypt, (C) South Africa, and (D) Algeria.

Estimated obesity prevalence was lowest for Ethiopia and highest for Libya during the study period (Figure 1). Obesity prevalence was most statistically significantly correlated with similar and different search terms across the countries with highest obesity and overweight prevalence (Figure 2). For example, for Libya, statistically significant correlations were observed between obesity prevalence and searches for yoga (*r*=0.95, 95% CI 0.71-0.99), exercise (*r*=0.89, 95% CI 0.43-0.98), and gym (*r*=0.91, 95% CI 0.49-0.99). Similarly, for Egypt, significant correlations were observed between obesity prevalence and searches for gym (*r*=0.98, 95% CI 0.83-0.99), breakfast (*r*=0.96, 95% CI 0.73-0.99), and yoga (*r*=0.95, 95% CI 0.67-0.99). In contrast, significant correlations for South Africa were between obesity prevalence and searches for how to exercise (*r*=0.99, 95% CI 0.91-0.99), green tea (*r*=0.98, 95% CI 0.89-0.99), and weight gain (*r*=0.97, 95% CI 0.83-0.99). For Algeria, we observed significant correlations between obesity prevalence and searches for gym (*r*=0.93, 95% CI 0.58-0.99), yoga (*r*=0.92, 95% CI 0.54-0.99), and weight (*r*=0.89, 95% CI 0.44-0.98). Searches for Fitbit were significantly associated with obesity prevalence in some countries (eg, Egypt and Algeria); however, the search volume was much lower than the search volume of other terms listed, suggesting less interest. Findings were similar between overweight prevalence and the search terms.

### Estimating Obesity With Search Trends

**Figure 3:**
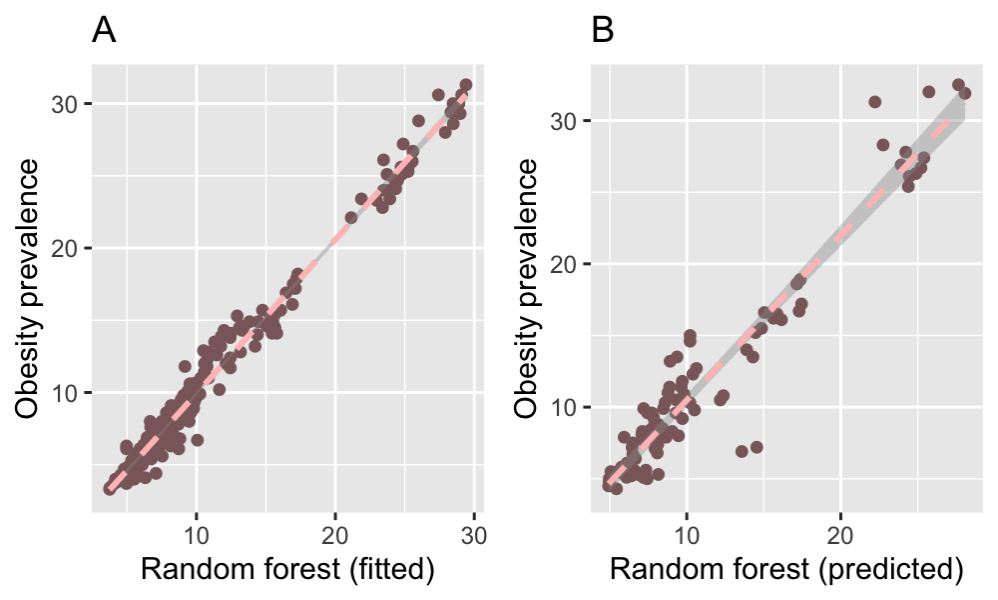
Estimation of obesity prevalence using search data and the random forest algorithm. (A) Association between model-estimated obesity prevalence and World Health Organization (WHO) obesity prevalence. (B) Association between model-predicted obesity prevalence and WHO obesity prevalence. The decision tree approaches had the lowest errors in estimating obesity prevalence.

Twelve of the terms that were significantly correlated with obesity prevalence (ie, hypertension, breakfast, diet, nutrition, obese, green tea, weight gain, lose weight, weight loss, weight, gym, and malnutrition) were used in modeling to estimate obesity prevalence. The estimated variances explained by the various models were 0.97, 0.92, 0.77, and 0.30 for RF (Figure 3), gradient boosting, SVM, and Bayes GLM, respectively; the corresponding RMSEs were 1.15, 1.87, 3.53, and 5.60, respectively. Likewise, the correlations between the out-of-sample estimates (ie, data not used to train the model) and obesity prevalence were 0.96, 0.94, 0.87, and 0.56 for RF, gradient boosting, SVM, and Bayes GLM, respectively.

**Figure 4:**
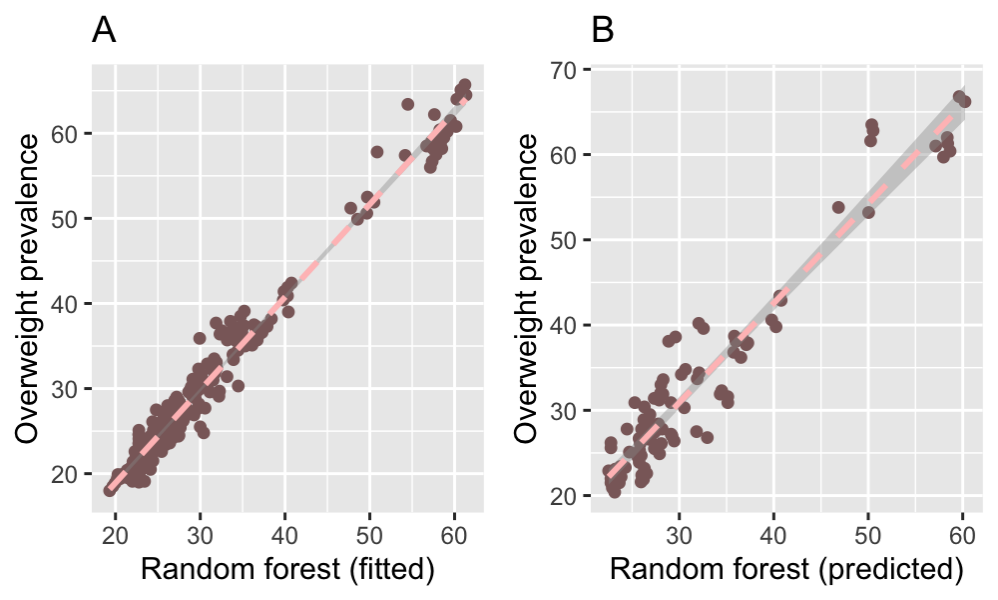
Estimation of overweight prevalence using search data and the random forest algorithm. (A) Association between model-estimated overweight prevalence and World Health Organization (WHO) overweight prevalence. (B) Association between model-predicted overweight prevalence and WHO overweight prevalence. The decision tree algorithms had the most accurate estimates of overweight prevalence.

Similarly, 8 search terms (hypertension, breakfast, diet, nutrition, obese, lose weight, gym, and malnutrition) were used in modeling to estimate overweight prevalence. The RF model was also the best performing model for estimating overweight prevalence (Figure 4). The estimated variances explained by the various models were 0.96 (RMSE 2.26), 0.91 (RMSE 3.56), 0.62 (RMSE 7.72), and 0.23 (RMSE 9.99) for RF, gradient boosting, SVM, and Bayes GLM, respectively; the corresponding correlations between the out-of-sample model estimates and overweight prevalence were 0.95, 0.94, 0.78, and 0.49, respectively.

## Discussion

Our study assessed the potential use of information-seeking trends of obesity- and overweight-related terms for monitoring these conditions in Africa. Several of the search terms were correlated with changes in obesity and overweight prevalence and, when modeled together, produced estimates that were significantly correlated with data from the WHO. Data from internet sources, including social media and search engines, can capture detailed information on individuals' well-being that can collectively reflect community perceptions of health. Web searches, unlike social media, can more accurately reflect information-seeking patterns on sensitive or stigmatized health topics since individuals tend to consider it private [55].

As African nations become more urbanized, digital data and tools could be useful for monitoring changes in behavioral risk factors, which could help public health officers, policy makers, health providers, and nutritionists to make informed decisions on chronic disease prevention efforts in Africa. Similarly, health care professionals can also use digital platforms to seek information on advances in medical practice, disseminate health information, and communicate with and support patients [56,57]. However, digital health implementation in some African countries is constricted by systemic hurdles such as weak health systems and a lack of coordination of mushrooming pilot projects [58].

A research agenda around monitoring risk factors for noncommunicable diseases using digital platforms should focus on quantifying changes with the intent to participate in behavioral risk factors, postings of engagement on social media, and information seeking on poor diet, physical inactivity, and other risk factors. Interventions can target younger populations—who tend to use digital platforms and are at risk—to promote healthy behaviors (eg, to stop smoking or reduce intake of sugary drinks). By monitoring changes in discussion trends on digital platforms, interventions designed for both online and offline targeting could be more beneficial, thereby avoiding the unintended effects of poorly designed campaigns. Furthermore, in regions where large data sets are available, systems can be developed for quantifying the prevalence of these risk factors at a granular level (ie, subnational or subregional)—using a combination of digital data, hospital data, and demographic data—where survey estimates are unavailable or delayed.

A major limitation of this study is that we did not collect data in other languages spoken in Africa (including Swahili, Portuguese, Sesotho, Zulu, Afrikaans, Xhosa, Tswana, Hausa, Tsonga, Afar, French, Arabic, and Somali). However, other studies suggest that English is used on the internet in many African countries [31,45]. Also, the obesity and overweight data are estimates that might not accurately reflect current obesity rates due to limitations in data and methods. Furthermore, the differences in search patterns between countries suggest a need for country-specific analysis. For example, there are local dieting fads (such as herbal life in South Africa) that should be monitored to capture local context. However, the number of observations was insufficient for fitting individual models to each country. Additionally, access to the internet might be influenced by socioeconomic status, which means that individuals seeking information on Google might not be representative of the total population [59-61].

However, our approach demonstrates that the adoption of internet technologies in Africa provides opportunities for studying and improving health. Obesity and overweight are health challenges faced by countries in Africa, and population information-seeking behaviors can inform how we design interventions. Information-seeking patterns on obesity-related risk factors could capture changes in attitudes, behaviors, and risk factor prevalence that could supplement official estimates from surveys.

## Appendix

Multimedia Appendix 1 A list of terms used to extract data from Google.

## Abbreviations

CI: credible interval
GLM: generalized linear model
RF: random forest
RMSE: root-mean-square error
STEPS: STEPwise approach to surveillance
SVM: support vector machine
WHO: World Health Organization

## References

[1] Stevens GA, Singh GM, Lu Y, Danaei G, Lin JK, Finucane MM, Bahalim AN, McIntire RK, Gutierrez HR, Cowan M, Paciorek CJ, Farzadfar F, Riley L, Ezzati M, Global Burden of Metabolic Risk Factors of Chronic Diseases Collaborating Group (Body Mass Index) (2012). National, regional, and global trends in adult overweight and obesity prevalences. Popul Health Metr.

[2] Lim SS, Vos T, Flaxman AD, Danaei G, Shibuya K, Adair-Rohani H, Amann Markus, Anderson H Ross, Andrews Kathryn G, Aryee Martin, Atkinson Charles, Bacchus Loraine J, Bahalim Adil N, Balakrishnan Kalpana, Balmes John, Barker-Collo Suzanne, Baxter Amanda, Bell Michelle L, Blore Jed D, Blyth Fiona, Bonner Carissa, Borges Guilherme, Bourne Rupert, Boussinesq Michel, Brauer Michael, Brooks Peter, Bruce Nigel G, Brunekreef Bert, Bryan-Hancock Claire, Bucello Chiara, Buchbinder Rachelle, Bull Fiona, Burnett Richard T, Byers Tim E, Calabria Bianca, Carapetis Jonathan, Carnahan Emily, Chafe Zoe, Charlson Fiona, Chen Honglei, Chen H, Cheng Andrew Tai-Ann, Child Jennifer Christine, Cohen Aaron, Colson K Ellicott, Cowie Benjamin C, Darby Sarah, Darling Susan, Davis Adrian, Degenhardt Louisa, Dentener Frank, Des Jarlais Don C, Devries Karen, Dherani Mukesh, Ding Eric L, Dorsey E Ray, Driscoll Tim, Edmond Karen, Ali Suad Eltahir, Engell Rebecca E, Erwin Patricia J, Fahimi Saman, Falder Gail, Farzadfar Farshad, Ferrari Alize, Finucane Mariel M, Flaxman Seth, Fowkes Francis Gerry R, Freedman Greg, Freeman Michael K, Gakidou Emmanuela, Ghosh Santu, Giovannucci Edward, Gmel Gerhard, Graham Kathryn, Grainger Rebecca, Grant Bridget, Gunnell David, Gutierrez Hialy R, Hall Wayne, Hoek Hans W, Hogan Anthony, Hosgood H Dean, Hoy Damian, Hu Howard, Hubbell Bryan J, Hutchings Sally J, Ibeanusi Sydney E, Jacklyn Gemma L, Jasrasaria Rashmi, Jonas Jost B, Kan Haidong, Kanis John A, Kassebaum Nicholas, Kawakami Norito, Khang Young-Ho, Khatibzadeh Shahab, Khoo Jon-Paul, Kok Cindy, Laden Francine, Lalloo Ratilal, Lan Qing, Lathlean Tim, Leasher Janet L, Leigh James, Li Yang, Lin John Kent, Lipshultz Steven E, London Stephanie, Lozano Rafael, Lu Yuan, Mak Joelle, Malekzadeh Reza, Mallinger Leslie, Marcenes Wagner, March Lyn, Marks Robin, Martin Randall, McGale Paul, McGrath John, Mehta Sumi, Mensah George A, Merriman Tony R, Micha Renata, Michaud Catherine, Mishra Vinod, Mohd Hanafiah Khayriyyah, Mokdad Ali A, Morawska Lidia, Mozaffarian Dariush, Murphy Tasha, Naghavi Mohsen, Neal Bruce, Nelson Paul K, Nolla Joan Miquel, Norman Rosana, Olives Casey, Omer Saad B, Orchard Jessica, Osborne Richard, Ostro Bart, Page Andrew, Pandey Kiran D, Parry Charles D H, Passmore Erin, Patra Jayadeep, Pearce Neil, Pelizzari Pamela M, Petzold Max, Phillips Michael R, Pope Dan, Pope C Arden, Powles John, Rao Mayuree, Razavi Homie, Rehfuess Eva A, Rehm Jürgen T, Ritz Beate, Rivara Frederick P, Roberts Thomas, Robinson Carolyn, Rodriguez-Portales Jose A, Romieu Isabelle, Room Robin, Rosenfeld Lisa C, Roy Ananya, Rushton Lesley, Salomon Joshua A, Sampson Uchechukwu, Sanchez-Riera Lidia, Sanman Ella, Sapkota Amir, Seedat Soraya, Shi Peilin, Shield Kevin, Shivakoti Rupak, Singh Gitanjali M, Sleet David A, Smith Emma, Smith Kirk R, Stapelberg Nicolas J C, Steenland Kyle, Stöckl Heidi, Stovner Lars Jacob, Straif Kurt, Straney Lahn, Thurston George D, Tran Jimmy H, Van Dingenen Rita, van Donkelaar Aaron, Veerman J Lennert, Vijayakumar Lakshmi, Weintraub Robert, Weissman Myrna M, White Richard A, Whiteford Harvey, Wiersma Steven T, Wilkinson James D, Williams Hywel C, Williams Warwick, Wilson Nicholas, Woolf Anthony D, Yip Paul, Zielinski Jan M, Lopez Alan D, Murray Christopher J L, Ezzati Majid, AlMazroa Mohammad A, Memish Ziad A (2012). A comparative risk assessment of burden of disease and injury attributable to 67 risk factors and risk factor clusters in 21 regions, 1990-2010: a systematic analysis for the Global Burden of Disease Study 2010. Lancet.

[3] Tydeman-Edwards R, Van Rooyen FC, Walsh CM (2018). Obesity, undernutrition and the double burden of malnutrition in the urban and rural southern Free State, South Africa. Heliyon.

[4] NCD Risk Factor Collaboration (NCD-RisC) – Africa Working Group (2017). Trends in obesity and diabetes across Africa from 1980 to 2014: an analysis of pooled population-based studies. Int J Epidemiol.

[5] Klingberg S, Draper C, Micklesfield L, Benjamin-Neelon S, van Sluijs E (2019). Childhood Obesity Prevention in Africa: A Systematic Review of Intervention Effectiveness and Implementation. Int J Environ Res Public Health.

[6] Neupane S, Prakash K C, Doku DT (2016). Overweight and obesity among women: analysis of demographic and health survey data from 32 Sub-Saharan African Countries. BMC Public Health.

[7] Ozodiegwu ID, Littleton MA, Nwabueze C, Famojuro O, Quinn M, Wallace R, Mamudu HM (2019). A qualitative research synthesis of contextual factors contributing to female overweight and obesity over the life course in sub-Saharan Africa. PLoS One.

[8] World Health Organzation Prevalence of obesity among adults, BMI ≥ 30, age-standardized estimates by WHO region. WHO.

[9] World Health Organization Prevalence of overweight among adults, BMI ≥ 25, age-standardized estimates by WHO Region. WHO.

[10] Mayosi BM (2013). The 10 'Best Buys' to combat heart disease, diabetes and stroke in Africa. Heart.

[11] Otang-Mbeng W, Otunola GA, Afolayan AJ (2017). Lifestyle factors and co-morbidities associated with obesity and overweight in Nkonkobe Municipality of the Eastern Cape, South Africa. J Health Popul Nutr.

[12] Engle-Stone Reina, Nankap M, Ndjebayi AO, Friedman A, Tarini A, Brown KH, Kaiser L (2018). Prevalence and predictors of overweight and obesity among Cameroonian women in a national survey and relationships with waist circumference and inflammation in Yaoundé and Douala. Matern Child Nutr.

[13] Mkuu RS, Epnere K, Chowdhury MAB (2018). Prevalence and Predictors of Overweight and Obesity Among Kenyan Women. Prev Chronic Dis.

[14] Adeboye B, Bermano G, Rolland C (2012). Obesity and its health impact in Africa: a systematic review. Cardiovasc J Afr.

[15] Steyn NP, McHiza Zandile J (2014). Obesity and the nutrition transition in Sub-Saharan Africa. Ann N Y Acad Sci.

[16] Toselli S, Gualdi-Russo E, Boulos DNK, Anwar WA, Lakhoua C, Jaouadi I, Khyatti M, Hemminki K (2014). Prevalence of overweight and obesity in adults from North Africa. Eur J Public Health.

[17] Monteiro C, Moura E, Conde W, Popkin B (2004). Socioeconomic status and obesity in adult populations of developing countries: a review. Bull World Health Organ.

[18] Mokhtar N, Elati J, Chabir R, Bour A, Elkari K, Schlossman N, Caballero B, Aguenaou H (2001). Diet culture and obesity in northern Africa. J Nutr.

[19] El Rhazi K, Nejjari C, Zidouh A, Bakkali R, Berraho M, Barberger Gateau P (2011). Prevalence of obesity and associated sociodemographic and lifestyle factors in Morocco. Public Health Nutr.

[20] Idung AU, Abasiubong F, Udoh SB, Ekanem US (2014). Overweight and obesity profiles in Niger Delta Region, Nigeria. Afr J Prim Health Care Fam Med.

[21] Lartey ST, Si L, de Graaff B, Magnussen CG, Ahmad H, Campbell J, Biritwum RB, Minicuci N, Kowal P, Palmer AJ (2019). Evaluation of the Association Between Health State Utilities and Obesity in Sub-Saharan Africa: Evidence From World Health Organization Study on Global AGEing and Adult Health Wave 2. Value Health.

[22] DeBono NL, Ross NA, Berrang-Ford L (2012). Does the Food Stamp Program cause obesity? A realist review and a call for place-based research. Health Place.

[23] Pi-Sunyer X (2009). The medical risks of obesity. Postgrad Med.

[24] Guh DP, Zhang W, Bansback N, Amarsi Z, Birmingham CL, Anis AH (2009). The incidence of co-morbidities related to obesity and overweight: a systematic review and meta-analysis. BMC Public Health.

[25] Joubert J, Norman R, Bradshaw D, Goedecke JH, Steyn NP, Puoane T, South African Comparative Risk Assessment Collaborating Group (2007). Estimating the burden of disease attributable to excess body weight in South Africa in 2000. S Afr Med J.

[26] Baleta A, Mitchell F (2014). Country in Focus: Diabetes and obesity in South Africa. Lancet Diabetes Endocrinol.

[27] Kengne AP, Echouffo-Tcheugui J, Sobngwi E, Mbanya J (2013). New insights on diabetes mellitus and obesity in Africa-part 1: prevalence, pathogenesis and comorbidities. Heart.

[28] Atun R, Gale EAM (2015). The challenge of diabetes in sub-Saharan Africa. Lancet Diabetes Endocrinol.

[29] Mbanya JC, Assah FK, Saji J, Atanga EN (2014). Obesity and type 2 diabetes in Sub-Sahara Africa. Curr Diab Rep.

[30] Petrakis D, Margină D, Tsarouhas K, Tekos F, Stan M, Nikitovic D, Kouretas D, Spandidos D, Tsatsakis A (2020). Obesity ‑ a risk factor for increased COVID‑19 prevalence, severity and lethality (Review). Mol Med Rep.

[31] Abebe Rediet, Hill Shawndra, Vaughan JW, Small PM, Schwartz HA (2019). Using Search Queries to Understand Health Information Needs in Africa. https://www.aaai.org/ojs/index.php/ICWSM/article/view/3360.

[32] Cesare N, Nguyen QC, Grant C, Nsoesie EO (2019). Social media captures demographic and regional physical activity. BMJ Open Sport Exerc Med.

[33] Cesare N, Dwivedi P, Nguyen QC, Nsoesie EO (2019). Use of Social Media, Search Queries, and Demographic Data to Assess Obesity Prevalence in the United States. Palgrave Commun.

[34] Culotta A (2010). Towards detecting influenza epidemics by analyzing Twitter messages.

[35] Santillana M, Nguyen AT, Dredze M, Paul MJ, Nsoesie EO, Brownstein JS (2015). Combining Search, Social Media, and Traditional Data Sources to Improve Influenza Surveillance. PLoS Comput Biol.

[36] Majumder MS, Kluberg S, Santillana M, Mekaru S, Brownstein JS (2015). 2014 ebola outbreak: media events track changes in observed reproductive number. PLoS Curr.

[37] Salathé Marcel, Bengtsson L, Bodnar TJ, Brewer DD, Brownstein JS, Buckee C, Campbell EM, Cattuto C, Khandelwal S, Mabry PL, Vespignani A (2012). Digital epidemiology. PLoS Comput Biol.

[38] Bakker KM, Martinez-Bakker ME, Helm B, Stevenson TJ (2016). Digital epidemiology reveals global childhood disease seasonality and the effects of immunization. Proc Natl Acad Sci U S A.

[39] Nsoesie E, Butler P, Ramakrishnan N, Mekaru S, Brownstein J (2015). Monitoring disease trends using hospital traffic data from high resolution satellite imagery: a feasibility study. Sci Rep.

[40] Nsoesie EO, Oladeji O, Sengeh MD (2020). Digital platforms and non-communicable diseases in sub-Saharan Africa. Lancet Digit Health.

[41] Nsoesie E, Oladeji O, Abah A, Ndeffo-Mbah M (2021). Forecasting influenza-like illness trends in Cameroon using Google Search Data. Sci Rep.

[42] Maharana A, Nsoesie EO (2018). Use of Deep Learning to Examine the Association of the Built Environment With Prevalence of Neighborhood Adult Obesity. JAMA Netw Open.

[43] Jalal M, Wang K, Jefferson S, Zheng Y, Nsoesie E, Betke M (2019). Scraping social media photos posted in Kenya and elsewhere to detect and analyze food types.

[44] Google Trends.

[45] Portland. How Africa Tweets 2015 Internet.

[46] WHO Prevalence of obesity among adults, BMI ≥ 30, age-standardized estimates by country.

[47] WHO Prevalence of overweight among adults, BMI ≥ 25, age-standardized estimates by country.

[48] NCD Risk Factor Collaboration (NCD-RisC) (2017). Worldwide trends in body-mass index, underweight, overweight, and obesity from 1975 to 2016: a pooled analysis of 2416 population-based measurement studies in 128·9 million children, adolescents, and adults. Lancet.

[49] WHO Obesity: preventing and managing the global epidemic.

[50] R Core team (2013). The R Project for Statistical Computing.

[51] Polley E, LeDell E, Kennedy C, Van DLM (2019). SuperLearner: Super Learner Prediction.

[52] Seeger M, Gerwinn S, Bethge M, Kok JN, Koronacki J, Mantaras RL, Matwin S, Mladenič D, Skowron A (2007). Bayesian Inference for Sparse Generalized Linear Models. Machine Learning: ECML 2007. ECML 2007. Lecture Notes in Computer Science, vol 4701.

[53] (2011). Bayesian Generalized Linear Models in R. Starkweather J.

[54] Kuhn M caret: Classification and Regression Training.

[55] De Choudhury M, Morris MR, White RW (2014). Seeking and sharing health information online: comparing search engines and social media. Proceedings of the SIGCHI Conference on Human Factors in Computing Systems.

[56] Kahn JG, Yang JS, Kahn JS (2010). 'Mobile' health needs and opportunities in developing countries. Health Aff (Millwood).

[57] Arigo D, Jake-Schoffman DE, Wolin K, Beckjord E, Hekler EB, Pagoto SL (2019). The history and future of digital health in the field of behavioral medicine. J Behav Med.

[58] Olu O, Muneene D, Bataringaya JE, Nahimana M, Ba H, Turgeon Y, Karamagi HC, Dovlo D (2019). How Can Digital Health Technologies Contribute to Sustainable Attainment of Universal Health Coverage in Africa? A Perspective. Front Public Health.

[59] Nsoesie EO, Flor L, Hawkins J, Maharana A, Skotnes T, Marinho F, Brownstein JS (2016). Social Media as a Sentinel for Disease Surveillance: What Does Sociodemographic Status Have to Do with It?. PLoS Curr.

[60] Henly S, Tuli G, Kluberg SA, Hawkins JB, Nguyen QC, Anema A, Maharana A, Brownstein JS, Nsoesie EO (2017). Disparities in digital reporting of illness: A demographic and socioeconomic assessment. Prev Med.

[61] Cesare N, Grant C, Hawkins J, Brownstein J, Nsoesie EO Demographics in Social Media Data for Public Health Research: Does it matter?.

