# Supplementary material for: Monitoring Information-Seeking Patterns and Obesity Prevalence in Africa With Internet Search Data: Observational Study
Source: JMIR Public Health Surveill. 2021 Apr 29;7(4):e24348. doi: 10.2196/24348 (PMC8120431; doi:10.2196/24348)
Supplement: Multimedia Appendix 1 [file publichealth_v7i4e24348_app1.pdf]

Monitoring Behavioral Risk Factors for Chronic Diseases in Africa with Internet Search Data

Olubusola Oladeji,[1] Chi Zhang,[2] Tiam Moradi,[2] Dharmesh Tarapore,[3] Andrew Stokes,[1] Vukosi Marivate,[4] Moinina David Sengeh,[5] Elaine O. Nsoesie[1*]

1. Department of Global Health, Boston University School of Public Health, Boston, MA, USA
2. Department of Computer Science, Boston University, Boston, MA, USA
3. BU Spark!, Hariri Institute for Computing, Boston University, Boston, MA, USA
4. Department of Computer Science, University of Pretoria, Pretoria, South Africa
5. Directorate of Science, Technology and Innovation, Freetown, Sierra Leone

* Corresponding author: Elaine O. Nsoesie, Department of Global Health, Boston University School of Public Health, 801 Massachusetts Ave, Crosstown Center 3rd Floor, Boston, MA 02119; onelaine@bu.edu; Tel: 617 358 3120

Supplementary Table S1. A list of terms used to extract data from Google.

Chronic Diseases and Conditions	Questions on Weight Loss and Obesity	Exercise, Diet and Obesity	Other Terms
Cardiovascular disease, Diabetes, Myocardial infarction, Obesity, Hypertension	does apple cider vinegar help to lose weight, how to drink apple cider vinegar to lose weight, how many calories should I eat to lose weight, how can I lose weight fast, how to get rid of belly fat, how to lose weight, what is obesity, how to lose weight while breastfeeding, how to eat healthy, how to exercise, where to buy keto weight loss plus, is obesity a disease, how to lose weight in 3 days, how to lose belly fat in one day, does green tea help with weight loss, how does intermittent fasting work	diet, exercise program, berberine weight loss, weight loss exercise program, fasting for weight loss, low-carbohydrate diet, belly fat burning, treadmill, weight loss pills, weight loss shakes, meal plan for weight loss, weight loss supplements, moringa weight loss, intermittent fasting for weight loss, best weight loss pill, obesity diet, gym, obesity exercise, exercise, diet pills, diet plan to lose weight, diet plan, low carb diet, low-fat diet, water fasting, ketogenic diet, low-calorie diet, very low-calorie diet, physical activity, very low calorie diet, low calorie, weight loss exercises, very-low calorie diet	malnutrition, bulimia, hypercholesterolemia, childhood obesity, adulthood obesity, abdominal obesity, management of obesity, obesity management, overweight, adipose tissue, anorexia, obesity risk, fat, weight gain, effects of obesity, five causes of obesity, body mass index, cause of obesity, nutrition, abdominal obesity, portion sizes, metabolism, tapeworm, lose weight, yoga, intermittent fasting, apple cider vinegar for weight loss recipe, ketovatru weight loss formula, best exercise for weight loss, eating plans for weight loss, chia seeds for weight loss, anti-obesity medication, ginger, breakfast, food, obesity breakfast, smoothies for weight loss, juicing for weight loss, fasting, liquid diet to lose weight, herbal life, fitbit, ketosis, pineapple, green tea, obesity disease, obesity stigma, obesity treatment, obesity research, obesity cure, obesity therapy, obese, obesity symptoms, how to eat healthy
